# Prognostic impact of TAZ and β-catenin expression in adenocarcinoma of the esophagogastric junction

**DOI:** 10.1186/1746-1596-9-125

**Published:** 2014-07-16

**Authors:** Lidan Sun, Fei Chen, Wenna Shi, Lei Qi, Zhongmei Zhao, Jianping Zhang

**Affiliations:** 1 Department of Pathology, Shandong University School of Medicine, 44#,Wenhua Xi Road, Jinan, Shandong 250012, People's Republic of China; 2 Department of Pathology, Qilu Hospital, Shandong University, 107#,Wenhua Xi Road, Jinan, Shandong 250012, People's Republic of China; 3 Department of Gastroenterology, Qilu Hospital, Shandong University, 107#,Wenhua Xi Road, Jinan, Shandong 250012, People's Republic of China; 4 Department of Orthopedics, Qilu Hospital, Shandong University, 107#,Wenhua Xi Road, Jinan, Shandong 250012, People's Republic of China; 5 Department of Gynecology and Obstetrics, Laiyang City Centre Hospital, Laiyang, Shandong 265200, People's Republic of China

**Keywords:** TAZ, β-catenin, Correlation, Adenocarcinoma of the esophagogastric junction, Esophagogastric junction, Prognosis, Immunohistochemistry

## Abstract

**Abstract:**

**Background**

TAZ is a downstream agent of Hippo signal pathway. β-catenin is a cell adhesion molecule associated with the invasion and metastasis of carcinomas as well as a critical component of Wnt pathway. TAZ and β-catenin have long been thought to play a vital role in tumour development and progression. This study aimed to detect expression of TAZ and β-catenin in adenocarcinoma of the esophagogastric junction (AEG) and explore their clinicopathological significance.

**Methods**

The expression of TAZ and β-catenin were detected by immunohistochemistry of 135 AEG samples, and analyzed with complete clinicopathological features. Overall survival rates were also calculated using the Kaplan-Meier method. Cox proportional hazard model was performed to assess the prognostic values. 37 normal mucosa and 41 dysplasia samples of esophagogastric junction (EGJ) were studied comparably.

**Results**

TAZ protein showed a strictly nuclear staining pattern in AEG and dysplasia with IHC. Expression of TAZ was higher in dysplasia and AEG compared with normal mucosa (P < 0.001, 0.008). The positive expression rate of nuclear β-catenin was significantly higher in carcinoma and dysplasia than that in normal mucosa (P < 0.001, =0.046). Abnormal expression rate of membranous β-catenin in AEG was significantly higher than that in normal mucosa tissues and dysplasia (P = 0.001, 0.002). In AEG, over expression of TAZ was directly correlated with abnormal nuclear β-catenin expression (r = 0.298, P < 0.001) and membranous β-catenin (r = 0.202, P = 0.019). Patients with abnormal TAZ or β-catenin expression of AEG exhibited a shorter overall survival (OS) and lower overall survival rate than those with normal TAZ or β-catenin expression (P < 0.05). In addition, patients with abnormal expression of both TAZ and β-catenin exhibited worst overall survival. In multivariate survival analysis, abnormal expression of TAZ, TAZ & β-catenin (nuclear and membranous) and tumour differentiation were found to be independent prognostic factors related to OS of AEG patients.

**Conclusions**

Over expression of TAZ was associated with abnormal expression of β-catenin, which is correlated with poor prognosis of patients with AEG. Abnormal expression of TAZ and TAZ & β-catenin (nuclear and membranous) are independent prognostic factors, so targeting TAZ and β-catenin could prove to be a promising therapeutic strategy for the treatment of AEG.

**Virtual Slides:**

The virtual slide(s) for this article can be found here: http://www.diagnosticpathology.diagnomx.eu/vs/2558852841276335

## Background

Adenocarcinoma of the esophagogastric junction (AEG) is defined as carcinoma that crosses the esophagogastric junction (EGJ) line, irrespective of where the tumour epicenter is located, including both distal esophageal and proximal gastric carcinomas [1]. In recent decades, AEG has gradually become a research hotspot for its rising incidence and obscure causes [2,3]. The recognition of AEG is not unanimous for the underlying mechanisms are poorly understood. There are still many controversies in its definition, classification, diagnosis and treatment. Some scientists distinguished patients with EGJ tumours from those with gastric cancer [4], but some treated them as esophageal cancer [5] or gastric tumours [6]. Therefore, this study explores the features of AEG to provide some evidence for clinical diagnosis and treatment.

The transcriptional coactivator with PDZ-binding motif (TAZ), also called WWTR1 (WW-domain containing transcriptional regulator 1), was first reported as a 14-3-3 binding protein [7] which is very similar to Yes-associated protein (YAP). It is one of the downstream agents of Hippo pathway which plays an important role in maintaining organ size and the regulation of cell proliferation and apoptosis [8,9]. The core components of Hippo pathway are mammalian STE20-like protein kinase 1/2 [10], large tumour suppressor 1/2 [11] and their adaptor proteins: Salvador homologue 1 [12] and MOB kinase activator respectively. That is a kinase cassette and finally leads to the phosphorylation of TAZ and YAP. Phosphorylation of YAP and TAZ loses their biological activities and stimulates their ubiquitin-mediated proteolysis [13]. Consequently, TAZ losses the ability of interacting with a variety of transcription factors to regulate cell growth differentiation and apoptosis, including Runx2, PPAR, TBX5, TEADs, TTF-1, and PAX3 [14-19].

β-catenin was originally identified as a membrane component of the cadherin-mediated cell-cell adhesion system and it is now widely recognized as a critical element of the Wnt signal pathway [20]. In the absence of Wnt signals, β-catenin is located at the plasma membrane, linked to E-cadherin, and functions in cell–cell adhesion. Excess cytoplasmic β-catenin is sequestered in a protein complex comprised of glycogen synthase kinase 3β (GSK-3β), adenomatous polyposis coli (APC) and AXIN1 or AXIN2. APC tumour suppressor gene product regulates the level of β-catenin protein by cooperating with GSK-3β via phosphorylation of serine/threonine residues coded on exon 3 of the β-catenin gene [21,22]. This phosphorylation is followed by degradation of β-catenin through the ubiquitin-proteasome pathway [23]. Activated Wnt signalling inhibits the phosphorylation of β-catenin, thereby preventing its degradation. The impaired β-catenin degradation leads to an increase in cytoplasmic β-catenin and its translocation to the nucleus. Nuclear β-catenin forms heterodimers with members of the TCF family of transcription factors and activates genes containing TCP-binding sites [24]. In some human cancers, mutation of either the APC gene or the β-catenin gene itself leads to the accumulation of β-catenin within the cancer cells [21,25]. Nuclear β-catenin is significantly associated with the invasion and metastasis of human cancers, such as carcinomas of esophagus, stomach, colon and melanomas [26,27].

The relationship between β-catenin and TAZ remains controversial. Some scholars believe that in the cytoplasm, the coactivator TAZ inhibits the CK1-mediated phosphorylation of Dvl2 by competing with Casein Kinase1 for combining with Dvl2, thereby inhibiting Wnt/β-catenin signalling and promoting the degradation of β-catenin [28]. Inhibiting the expression of TAZ increases the levels of β-catenin and downstream effectors of Wnt pathway. But some are arguing that in the absence of Wnt activity, the components of the β-catenin destruction complex--APC, Axin, and GSK3--are also required to keep TAZ at low levels. TAZ degradation depends on phosphorylated β-catenin that bridges TAZ to its ubiquitin ligase β-TrCP. Upon Wnt signal, escape of β-catenin from the destruction complex impairs TAZ degradation and leads to concomitant accumulation of β-catenin and TAZ [29]. The activation of TAZ involves in Wnt signal pathway and mediates important biological effects [30]. The activated hippo pathway can reduce stability and transcriptional activity in nuclear of β-catenin by phosphorylation of YAP and TAZ [31].

Although β-catenin or TAZ has been demonstrated to correlate with poor prognosis of a variety of malignancies, however, the correlation of TAZ and β-catenin expression in AEG and the relevance of their co-expression within clinical parameters still remain unclear. Moreover, the relationship between β-catenin and TAZ remains controversial.

In this study, expression of TAZ and β-catenin was examined using immunohistochemistry on 135 AEG samples, compared with normal tissues and dysplasia samples. The correlation of TAZ and β-catenin expression and its relevance to clinicopathologic parameters were explored. Furthermore, the prognostic roles of TAZ and β-catenin in AEG were evaluated using Kaplan-Meier and Cox regression analysis. To the best of our knowledge, it is the first instance of reporting the correlation of TAZ and β-catenin expression and their clinical significance for patients with AEG.

## Methods

### Patients and tissue samples

The samples were obtained upon receipt of informed consent from patients undergoing surgical resection. The study was approved by the ethics committee of Shandong University School of Medicine (Jinan, China). Total samples comprised 135 cases of adenocarcinoma, 37 cases of normal mucosa tissues and 41 cases of dysplasia specimens which were located in EGJ. The cases were obtained from the archives of the department of Pathology at Qilu Hospital, Shandong University and collected from patients who underwent surgery between January 2006 and December 2007. The diagnosis was confirmed histologically in all cases, based mainly on examination of sections stained with H&E. Before surgery, no patients had received drug intervention and preoperative chemotherapy. Pathological characteristics were obtained from the medical records and the original pathology reports, including age, gender, tumour differentiation, tumour size, lymph node status and invasion of serosa. AEG cases were staged by TNM classification according to the standard for oesophagogastric junction tumours of American Joint Committee on Cancer (AJCC). Curative resection procedure was performed in all AEG patients and the resection boundaries were negative both in intraoperative and routine pathological diagnosis. The AEG patients were followed up by phone call. The total period of follow-up was 1–81 months (median was 35 months). The endpoint of this study was overall survival which is defined as the period lapsing from the date of initial biopsy until death or last follow. By October 2013, 89 patients were reached the end events.

### Immunohistochemical stains

Immunohistochemical stains were performed on formalin-fixed, paraffin-embedded specimens with the PV-9000 2-step plus poly-HRP anti-mouse/rabbit IgG detection system (ZSGB-Bio, Beijing, China) according to the manufacturer’s instructions. Briefly, 4 μm sections were cut and placed on glass slides, then dewaxed in xylene and rehydrated through graded alcohol. All the slides were boiled in EDTA antigen retrieval solution (pH 9.0, ZSGB-Bio, Beijing, China) for 3 min to retrieval the antigen. Thereafter, the sections were incubated with 3% hydrogen peroxidase (reagent A) at room temperature (RT) for 10 min to block endogenous peroxidase activity. After rinsing in phosphate buffered saline (PBS, PH 7.2), incubated with normal goat serum (ZSGB-Bio, Beijing, China) to block any nonspecific reactions for 10 min at RT. Shook off excess goat serum, and then incubated the slides with primary antibodies overnight at 4˚C, TAZ (bs-12367R, Bioss, Beijing, China) and β-catenin (ZM-0442, ZSGB-Bio, Beijing, China) were diluted 1:100 and 1:200 in PBS. The sections were washed with PBS and incubated with polymer helper (reagent B) for 20 min. After rinsing in PBS, the sections were incubated with polyperoxidase-anti-mouse/rabbit IgG (reagent C) for 30 min at room temperature and DAB (ZSGB-Bio, Beijing, China) was visualized. After rinsing in water, the sections were counterstained with hematoxylin, dehydrated, and coverslipped.

### Positive and negative control

Samples of AEG with high expression of TAZ and β-catenin served as the positive control. PBS was used instead of the primary antibodies as negative controls.

### Evaluation of immunostaining

TAZ and β-catenin immunostaining signals were evaluated independently by two pathologists in a blinded manner. Brown nuclear staining for TAZ was considered positive/abnormal. The staining intensity of positive tumour cells was scored as 0 (no staining), 1 (weak staining), 2 (moderate staining) and 3 (strong staining). The percentage of positively stained tumour cells was scored with 5 scales: 0 (<10 %); 1 (10% to 25%); 2 (26% to 50%); 3 (51% to 75%); 4 (>75%). The final score was the product of the intensity and the percentage. For statistical reasons, a final staining score ≥5 was considered to be positive. Nuclear and membranous β-catenin signals were evaluated independently. When more than 10% of cancer cells showed strong nuclear staining, the tumour was judged to exhibit positive/abnormal nuclear expression. In cases that demonstrated either no immunoreactivity at the membrane, or less than 10% of the tumour cells with positive membranous staining, the specimen was considered to abnormal membranous expression [32]. The percentage of positively stained cells was estimated in an average of 100 cells counted in more than 5 high-power fields (×400).

### Statistical analysis

Kruskal-Wallis text was used to assess the difference of TAZ or β-catenin expression in the three groups. The relationship between TAZ or β-catenin expression and clinicopathologic parameters were analyzed using Chi-square test and *T* test. The correlation between TAZ and β-catenin was analyzed using the spearman’s rank test. The survival curves were estimated by the Kaplan–Meier method. Log-rank test was used to compare survival curves. The HR and the 95% CI were evaluated for each variable using the Cox univariate model. A multivariate Cox proportional hazard model was also developed using stepwise regression (forward selection) with predictive variables that were significant in the univariate analyses. P < 0.05 was considered to be statistically significant. All statistical analyses were carried out using SPSS software (SPSS version 17.0 SPSS, Inc., Chicago, IL, USA).

## Results

### Expression of TAZ and β-catenin

**Figure 1:**
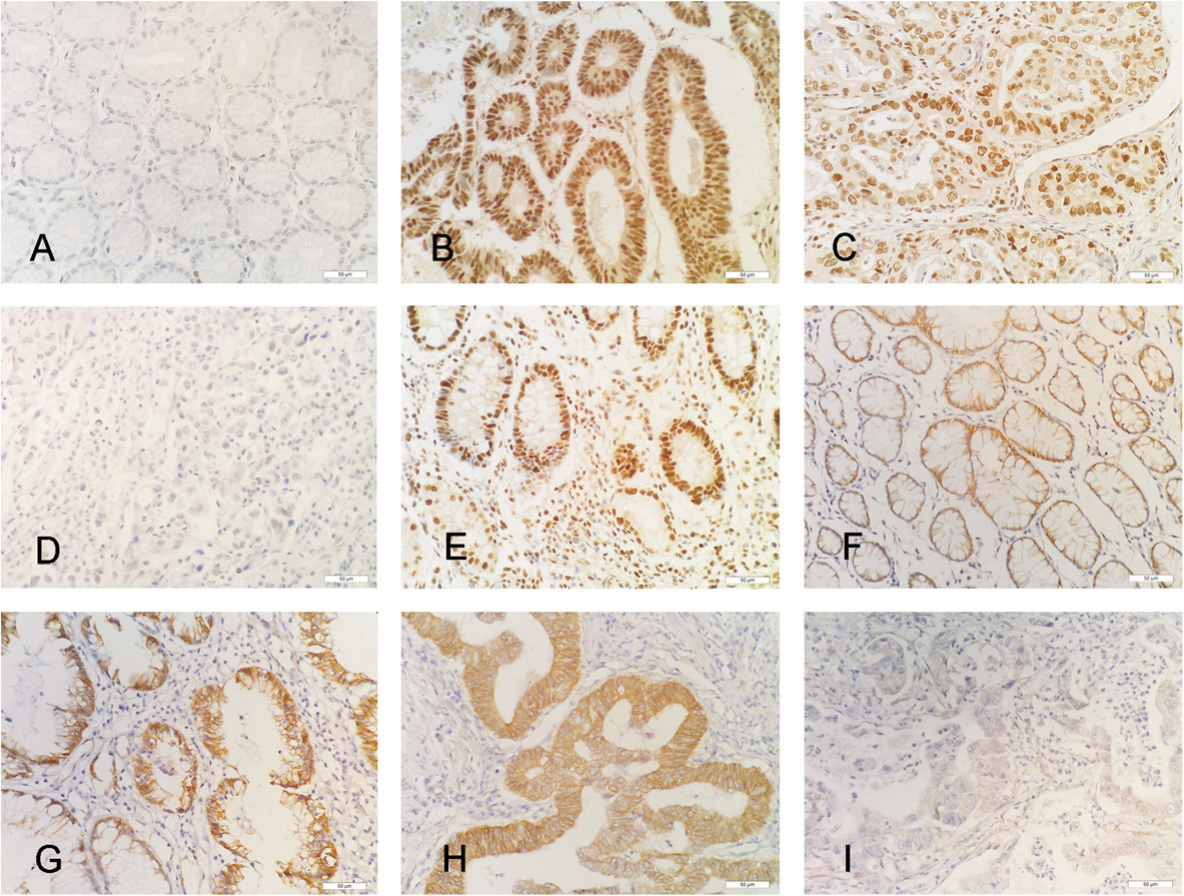
**Expression of TAZ and β-catenin in adenocarcinoma, dysplasia and normal mucosa samples of EGJ. (A)** TAZ-negative in normal mucosa. **(B)** TAZ-positive in dysplasia. **(C)** TAZ-positive in adenocarcinoma. **(D)** TAZ-negative in adenocarcinoma. **(E)** TAZ-positive in intestinal metaplasia. **(F)** Nuclear β-catenin negative and normal expression of membranous β-catenin in normal mucosa. **(G)** β-catenin- positive in dysplasia. **(H)** β-catenin- positive in adenocarcinoma. **(I)** Abnormal membranous expression of β-catenin in adenocarcinoma. (Original magnification, ×400).

As shown in Figure 1 and Table 1, a positive expression of TAZ was observed in normal mucosa 16.2% (6/37), dysplasia 70.7% (29/41), and AEG 40.7% (55/135) and the difference was significant (H = 23.922, P <0.001). The expression of TAZ in dysplasia and AEG is higher than that in normal mucosa (P < 0.001, =0.008). And it is worth reminding that the expression of TAZ in dysplasia is higher than that in AEG (P = 0.001).

**Table 1:** Expression of TAZ and β-catenin proteins in different disease of EGJ

	**Total**	**TAZ**		**Nuclear β-catenin**		**Membranous β-catenin**	
	**(n)**	**Positive (%)**	**Negative (%)**	**Positive (%)**	**Negative (%)**	**Abnormal (%)**	**Normal (%)**
**Normal mucosa**	37	6(16.2)	31(83.8)	7(18.9)	30(81.1)	10(27)	27(73)
**Dysplasia**	41	29^***^ (70.7)	12 (29.3)	17^*^ (41.5)	31(58.5)	12(29.3)	29(70.7)
**AEG**	135	55^**#^(40.7)	80 (59.3)	69^***^ (51.1)	66(48.9)	77^**#^(57)	58(43)

Compared with Normal mucosa: *P < 0.05, **P < 0.01, ***P < 0.001; compared with Dysplasia: ^#^P < 0.01.

The positive nuclear expression rates of β-catenin in normal mucosa, dysplasia and AEG were 18.9% (7/37), 41.5% (17/41) and 51.1% (69/135) and there was statistical difference among these groups (H = 12.277, P = 0.002). The expression of β-catenin in AEG and dysplasia were significantly higher than in normal mucosa (P < 0.001, =0.046) while no significant difference between AEG and dysplasia (P = 0.276).

The abnormal membranous expression rates of β-catenin in the normal mucosa, dysplasia and AEG were 27% (10/37), 29.3% (12/41) and 57% (77/135), respectively. There was a significant statistical difference among the groups (H = 16.482, P < 0.001). Abnormal membranous expression of β-catenin in AEG was significantly higher than that in normal mucosa tissues and dysplasia (P = 0.001, 0.002), while no difference was found between normal mucosa tissues and dysplasia (P = 0.843).

In AEG, interestingly, the statistical analysis revealed that TAZ expression positively correlated with nuclear β-catenin expression (r = 0.298; confidence interval (CI) 95%, 0.136-0.462; P < 0.001) (Table 2). In a separate analysis, abnormal membranous expression of β-catenin is also correlated with expression of TAZ (r = 0.202; CI 95%, 0.041-0.357; P = 0.019).

**Table 2:** Relationship between the expression of TAZ and β-catenin in AEG

**TAZ**	**Membranous β-catenin**		**Nuclear β-catenin**	
	**Normal**	**Abnormal**	**Negative**	**Positive**
**Negative**	41	39	49	31
**Positive**	17	38	17	38
	r = 0.202; P = 0.019^*^		r = 0.298; P < 0.001^*^	

*Statistically significant.

### Association of TAZ and β-catenin expression with clinicopathological characteristics

As shown in Table 3, the abnormal expression of TAZ, nuclear β-catenin, membranous β-catenin, TAZ & nuclear β-catenin and TAZ & membranous β-catenin were markedly correlated with lymph node metastasis, invasion of serosa and tumour differentiation (P < 0.05, respectively). In addition, the abnormal expression of nuclear β-catenin and TAZ & membranous β-catenin were positively correlated with age (P < 0.001, =0.037). The abnormal expression of TAZ, membranous β-catenin, TAZ & nuclear β-catenin and TAZ & membranous β-catenin were markedly correlated with TNM stages. There was no significant association of TAZ or β-catenin expression with other clinicopathological features mentioned in the table.

**Table 3:** Relationship between the clinicopathologic characteristics of AEG and the expression of TAZ or β-catenin

**Clinicopathologic characteristics**	**TAZ**		**P**	**β-catenin (nuclear)**		**P**	**β-catenin (membranous)**		**P**	**TAZ & β-catenin (nuclear)**		**P**	**TAZ & β-catenin (membranous)**		**P**
	**Negative**	**Positive**		**Negative**	**Positive**		**Normal**	**Abnormal**		**Others**	**Positive**		**Others**	**Positive**	
**Gender**															
Male	64	46	0.593	52	58	0.431	45	65	0.312	79	31	0.99	64	46	0.842
Female	16	9		14	11		13	12		18	7		14	11	
**Age**															
<60 years old	26	22	0.371	13	35	0.001	21	27	0.891	31	17	0.163	22	26	0.037
≥60 years old	54	33		53	34		37	50		66	21		56	31	
**Invasion of serosa**															
Positive	21	38	0.001	21	38	0.006	18	41	0.010	31	28	0.001	25	34	0.001
Negative	59	17		45	31		40	36		66	10		53	23	
**lymphatic metastasis**															
Positive	34	42	0.001	30	46	0.013	27	49	0.048	43	33	0.001	37	39	0.015
Negative	46	13		36	23		31	28		54	5		41	18	
**Differentiation**															
Well-differentiated	51	22	0.007	43	30	0.012	47	26	0.001	59	14	0.012	57	16	0.001
Poorly differentiated	29	33		23	39		11	51		38	24		21	41	
**TNM stage**															
Stage 0 I II	55	24	0.004	43	36	0.126	39	40	0.074	65	14	0.001	65	32	0.001
Stage III IV	25	31		23	33		19	37		32	24		14	24	

No significant correlations were found between expression of TAZ or β-catenin and tumour size. The tumour size of TAZ positive cases was 4.74 ± 1.872 centimeter, the negative one was 5.03 ± 1.923 centimeter, P = 0.392. The tumour sizes of nuclear β-catenin positive was 4.92 ± 2.019 centimeter and the negative one was 4.79 ± 1.686 centimeter, P = 0.685. The tumour sizes of abnormal expression membranous β-catenin was 4.89 ± 1.758 centimeter and the normal one was 4.83 ± 2.059 centimeter, P = 0.863.

### Association of TAZ and β-catenin expression with OS

**Figure 2:**
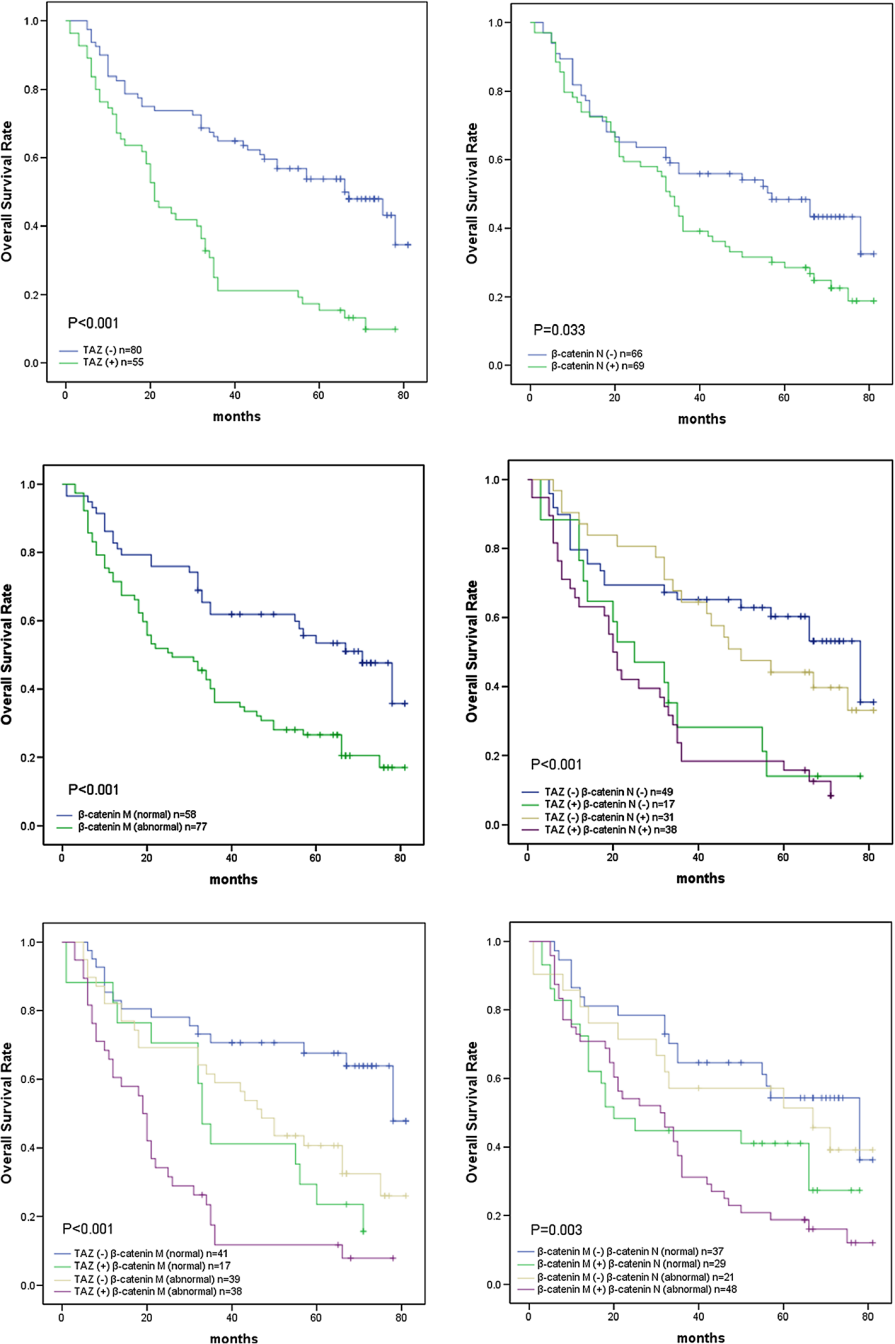
**Kaplan–Meier analysis of overall survival rates in 135 AEG patients.** β-catenin N, nuclear expression of β-catenin. β-catenin M, membranous expression of β-catenin.

**Figure 3:**
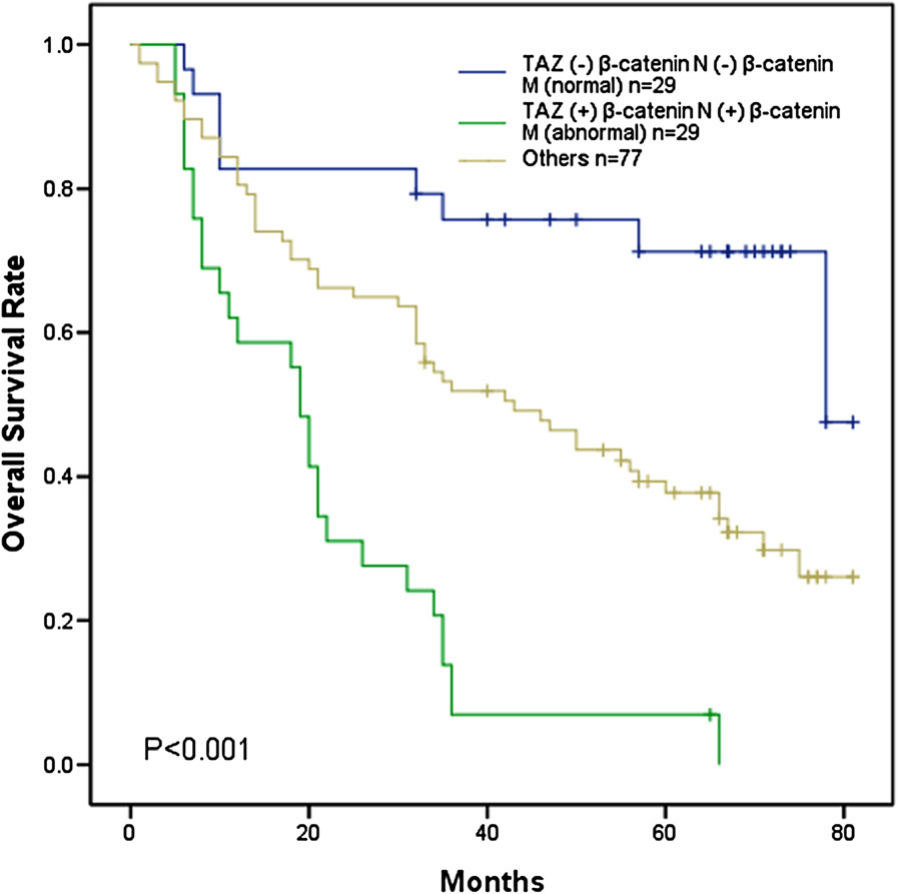
**Kaplan–Meier analysis of AEG patients with abnormal expression of TAZ & β-catenin (nuclear and membranous).** β-catenin N, nuclear expression of β-catenin. β-catenin M, membranous expression of β-catenin.

When the AEG cases were analyzed by Kaplan–Meier curves (Figure 2), we observed that the abnormal expression of TAZ, β-catenin (nuclear and membranous), nuclear β-catenin, membranous β-catenin, TAZ & nuclear β-catenin and TAZ & membranous β-catenin significantly affected overall survival rate of AEG patients (P < 0.05 for all). Patients with abnormal expression tumour exhibited a shorter OS than those with normal one. Moreover, patients with both TAZ and β-catenin (nuclear and membranous) abnormal expression exhibited the worst survival (mean of 21.2 ± 3.0 months and median of 19 months). On the contrary, patients with both normal expression demonstrated the best overall survival (mean of 63.4 ± 5.2 months and median of 78 months, P < 0.001, Figure 3). As summarized in Table 4, the Cox univariate model highlighted that abnormal expression of TAZ, nuclear β-catenin, membranous β-catenin, TAZ & nuclear β-catenin, TAZ & membranous β-catenin, TAZ & β-catenin (nuclear and membranous) tumour differentiation and nodal status significantly impact overall survival time (P < 0.05 for all). More importantly, multivariate analysis revealed that tumour differentiation (Hazard rate (HR) 1.870, CI 95%, 1.216-2.877, P = 0.004), TAZ expression (HR 1.879, CI 95%, 1.079-3.218, P = 0.022) and abnormal expression of TAZ & β-catenin (nuclear and membranous) (HR 1.899, CI 95%, 1.053-3.423, P = 0.033) were independent negative prognostic variables influencing OS.

**Table 4:** Cox proportional hazard regression model analysis

**Factors**	**Univariate analysis**		**Multivariate analysis**	
	**HR (95% CI)**	**P-value**	**HR (95% CI)**	**P-value**
**TAZ**				
Positive vs. Negative	2.740 (1.791-4.192)	<0.001^*^	1.879 (1.097-3.218)	0.022^*^
**β-catenin (nuclear)**				
Positive vs. Negative	1.572 (1.028-2.402)	0.037^*^		
**β-catenin (membranous)**				
Abnormal vs. Normal	2.153 (1.377-3.367)	<0.001^*^		
**TAZ & β-catenin (nuclear)**				
Positive vs. All others	2.482 (1.606-3.837)	<0.001^*^		
**TAZ & β-catenin (membranous)**				
Abnormal vs. All others	3.458 (1.732-6.908)	<0.001^*^		
**TAZ & β-catenin (nuclear and membranous)**				
Abnormal vs. All others	3.330 (2.082-5.328)	<0.001^*^	1.899 (1.053-3.423)	0.033^*^
**Tumor size (diameter)**				
≥ 3 cm vs. <3 cm	1.490 (0.886-2.506)	0.132		
**Lymph node status**				
Positive vs. Negative	1.548 (1.006-2.383)	0.047^*^		
**Tumor differentiation**				
Poorly vs. Well-differentiated	2.165 (1.419-3.304)	<0.001^*^	1.870 (1.216-2.877)	0.004^*^
**Age (years)**				
≥ 60 vs. < 60	1.216 (0.782-1.890)	0.386		
**Gender**				
Male vs. Female	1.687 (0.918-3.102)	0.092		
**Invation of serosa**				
Positive vs. Negative	1.355 (0.893-2.057)	0.154		

*Statistically significant.

## Discussion

AEG includes adenocarcinomas of distal esophageal, cardia and proximal gastric and has been regarded as a separate entity because it appears to have distinct features. Proximal gastric cancer, which is similar with distal esophageal adenocarcinoma, is very different from distal gastric cancer on patient’s age, gender, incidence, tumour biological behaviors and clinicopathological features [33]. Lower thoracic esophageal carcinoma is more likely to have lymph node metastasis, fast proliferation and poor prognosis than the upper or middle one. So the concept of AEG was put forward as a special entity. The incidence of AEG is going higher than that of distal stomach cancer [34]. The possible causes of AEG and gastric cancer are different. Notably, it has been demonstrated that H. pylori infection is one of the clear risk factors to gastric cancer, while some research suggests that negative H. pylori status correlates with a poor prognosis with AEG [35,36]. Gastric cancers located in the distal stomach are associated with better prognosis than those located in the proximal region [37]. Differences in sex distinction and mean age were found between AEG and the distal gastric cancer [38]. In our study, the ratio of man to woman was 4.4 to 1, and the average age was 62.6 years old, and patients of 60 years old and above were accounted for 64.4 percent of AEG patients, which were consistent with previous studies. The rate of lymph node metastasis was 56.3%, and associated with over expression of TAZ or β-catenin. Some earlier researches produced similar findings: β-catenin was over expressed in metastatic sentinel lymph node, and strongly associated with liver metastasis [39]. The percentage of well differentiated carcinoma in AEG (49.5%) was higher than that in other malignant tumours [40].

The accumulated evidence from basic and clinical researches indicates that both TAZ and β-catenin were correlated with tumour invasion, metastasis, and poor prognosis in human cancers [21,26,27,41,42]. However, the regulation of TAZ upon β-catenin expression and the relevance of their expression to clinical pathological features in AEG were still unknown. In this study, we examined the expression of TAZ and β-catenin in 135 AEG specimens using immunohistochemistry stain, and compared with expressions in normal mucosa and dysplasia samples. To our knowledge, it is the first time that clinical evidence has been provided to indicate that TAZ and β-catenin expression were positively correlated in AEG.

TAZ is a WW domain containing transcriptional coactivator that modulates cell differentiation and development of multiple organs [7]. TAZ promotes cell proliferation and epithelial-mesenchymal transition and plays an important role in tumourigenesis [43]. Zhao et al. reported that TAZ can promote cell growth in breast partially through up-regulating KLF5 protein and enhancing its activity and protecting it from WWP1-mediated degradation [44]. There was also a report that TAZ induces growth factor-independent proliferation through activation of EGFR ligand amphiregulin [45]. Here we found that TAZ protein was strictly located in the nucleus of AEG cells using IHC staining, and the expression level of TAZ was significantly upregulated in AEG and dysplasia than that in normal mucosa. Moreover, we also observed that the expression of TAZ was positive in intestinal metaplasia of EGJ (Figure 1E). These results suggest that TAZ upregulation is an early event in the progression of AEG.

β-catenin plays an essential role in the regulation of the E-cadherin-catenin cell adhesion complex as well as in the Wnt signal pathway [20]. It has been reported that β-catenin was significantly associated with the invasion and metastasis of carcinomas of the esophagus, stomach, colon, liver and melanomas [21,26,27,46]. In this study, we evaluated nuclear and membranous β-catenin signals respectively in immunohistochemical stain. Nuclear β-catenin expression in three groups was similar with TAZ, indicating that nuclear β-catenin accumulation was involved in the carcinogenesis and tumour development of AEG. While abnormal membranous expression rate of β-catenin in AEG was higher than that in normal mucosa and dysplasia, no difference between normal mucosa and dysplasia. Furthermore, abnormal expression of membranous β-catenin was correlated with lymph node matastisis and invasion of serosa. We can infer that the decreased expression of membranous β-catenin may occur in later stage of AEG and associated with invasion and metastasis.

In our study, the abnormal expression of TAZ, nuclear β-catenin, membranous β-catenin, TAZ & nuclear β-catenin and TAZ & membranous β-catenin were positively correlated with lymph node metastasis, invasion of serosa and tumour differentiation which are crucial histological features associated with poor prognosis, and these findings were consistent with previous results. Because TNM classification is closely related to lymph node status, tumour invasion and metastasis, the abnormal expression of TAZ, membranous β-catenin, TAZ & nuclear β-catenin and TAZ & membranous β-catenin were also markedly correlated with TNM stages. We believe that the abnormal TAZ or β-catenin expression in tumour cells promotes tumour cell EMT and, therefore, facilitates tumour cell migration and metastasis into the lymphatic vessels.

In this study over expression of TAZ and nuclear expression of β-catenin were positively correlated, which was consistent with the previous findings [29-31]. We can infer that except for acting as a transcription coactivator, TAZ may play a key role in suppressing the degradation of β-catenin and promoting β-catenin to enter nuclear region. We also found that the expression of TAZ was positively associated with decreased membranous expression of β-catenin. However, the molecular mechanism underlying this expression pattern and its clinical importance need future investigation. One possible mechanism is TAZ affects cadherin-mediated cell-cell adhesion system by regulating membranous expression of β-catenin.

Previously reported that colorectal cancer patients with higher TAZ expression showed a trend of shorter survival times [42]. Consistently, in our study the Kaplan–Meier survival analysis revealed that patients with abnormal expression of TAZ or β-catenin (nuclear or membranous) expression showed worse OS than those with normal TAZ or β-catenin expression. Further still, patients with abnormal expression of both TAZ and β-catenin (nuclear and membranous) exhibited the worst overall survival. On the contrary, patients with both normal results demonstrated the best survival. These results indicate that combining TAZ and β-catenin predicts worse survival and may serve as the key molecular prognostic indicator for AEG patient survival.

Consistent with previous reports, our Cox multivariate analysis demonstrated that high TAZ expression levels and combined abnormal expression of TAZ & β-catenin (nuclear and membranous) were independent negative prognostic factors together with tumour differentiation. The result strongly suggests that TAZ and β-catenin may serve as disease prognosis indicators for AEG patient survival. Moreover, the regulation of TAZ on β-catenin expression provides a new molecular mechanism underlying TAZ promoted AEG progression and metastasis and may indeed suggest a feasible therapeutic strategy to inhibit AEG metastasis by targeting TAZ and β-catenin expressions.

## Conclusions

In conclusion, our research demonstrated that over expression of TAZ was associated with abnormal expression of β-catenin in AEG. We also provided convincing evidence that abnormal expression of TAZ or β-catenin was correlated with the poor prognosis of patients with AEG. Moreover, abnormal expression of TAZ and TAZ & β-catenin (nuclear and membranous) could be independent predictors of prognosis in AEG, so targeting TAZ and β-catenin could prove to be a promising therapeutic strategy for the treatment of AEG.

## Abbreviations

AEG: Adenocarcinoma of the esophagogastric junction; EGJ: Esophagogastric junction; TAZ: Transcriptional coactivator with PDZ-binding motif; CI: Confidence interval; HR: Hazard ratio; OS: Overall survival.

## Competing interests

The authors declared that they have no competing interests.

## Authors’ contributions

ZJ conceived of the study. SL participated in experiments, performed the statistical analysis and drafted the manuscript. CF and SW participated in case selection and followed up with AEG patients. All authors scored the immunostaining, read and approved the final manuscript.
